# MRI-Seed-Wizard: combining deep learning algorithms with magnetic resonance imaging enables advanced seed phenotyping

**DOI:** 10.1093/jxb/erae408

**Published:** 2024-10-09

**Authors:** Iaroslav Plutenko, Volodymyr Radchuk, Simon Mayer, Peter Keil, Stefan Ortleb, Steffen Wagner, Volker Lehmann, Hardy Rolletschek, Ljudmilla Borisjuk

**Affiliations:** Leibniz-Institute of Plant Genetics and Crop Plant Research (IPK), Corrensstrasse 3, 06466 Seeland OT Gatersleben, Germany; Leibniz-Institute of Plant Genetics and Crop Plant Research (IPK), Corrensstrasse 3, 06466 Seeland OT Gatersleben, Germany; Leibniz-Institute of Plant Genetics and Crop Plant Research (IPK), Corrensstrasse 3, 06466 Seeland OT Gatersleben, Germany; Institute of Experimental Physics 5, University of Würzburg, Am Hubland, 97074 Würzburg, Germany; Leibniz-Institute of Plant Genetics and Crop Plant Research (IPK), Corrensstrasse 3, 06466 Seeland OT Gatersleben, Germany; Leibniz-Institute of Plant Genetics and Crop Plant Research (IPK), Corrensstrasse 3, 06466 Seeland OT Gatersleben, Germany; Leibniz-Institute of Plant Genetics and Crop Plant Research (IPK), Corrensstrasse 3, 06466 Seeland OT Gatersleben, Germany; Bruker BioSpin GmbH, Rudolf-Plank-Str. 23, 76275 Ettlingen, Germany; Leibniz-Institute of Plant Genetics and Crop Plant Research (IPK), Corrensstrasse 3, 06466 Seeland OT Gatersleben, Germany; Leibniz-Institute of Plant Genetics and Crop Plant Research (IPK), Corrensstrasse 3, 06466 Seeland OT Gatersleben, Germany; MPI of Molecular Plant Physiology, Germany

**Keywords:** Automatic segmentation, deep learning, grain phenotyping, magnetic resonance imaging, morphometry, wheat

## Abstract

Evaluation of relevant seed traits is an essential part of most plant breeding and biotechnology programmes. There is a need for non-destructive, three-dimensional assessment of the morphometry, composition, and internal features of seeds. Here, we introduce a novel tool, MRI-Seed-Wizard, which integrates deep learning algorithms with non-invasive magnetic resonance imaging (MRI) for use in a new domain—plant MRI. The tool enabled *in vivo* quantification of 23 grain traits, including volumetric parameters of inner seed structure. Several of these features cannot be assessed using conventional techniques, including X-ray computed tomography. MRI-Seed-Wizard was designed to automate the manual processes of identifying, labeling, and analysing digital MRI data. We further provide advanced MRI protocols that allow the evaluation of multiple seeds simultaneously to increase throughput. The versatility of MRI-Seed-Wizard in seed phenotyping is demonstrated for wheat (*Triticum aestivum*) and barley (*Hordeum vulgare*) grains, and it is applicable to a wide range of crop seeds. Thus, artificial intelligence, combined with the most versatile imaging modality, MRI, opens up new perspectives in seed phenotyping and crop improvement.

## Teaser

Deep learning coupled with magnetic resonance imaging enables *in vivo* phenotyping of seeds and unveils previously inaccessible internal seed traits.

## Introduction

Seeds form the foundation of agriculture and hold the key to delivering food, fuel, and fiber needed to sustain the global population. Quantitative assessment and understanding of seed traits are necessary to improve plant performance under rapidly evolving climatic conditions. Comprehensive seed phenotyping is highly relevant for most breeding and biotechnology programmes. Most screening tools target either the composition or the external morphometric parameters of seeds, such as seed shape, length, or width (Liu *et al*., 2024). Widely used non-invasive commercial and scientific tools such as SmartGrain (Tanabata *et al*., 2012) and the MARVIN analyser (Gegas *et al*., 2010; Escudero-Martinez *et al*., 2022; He *et al*., 2022; Jayakodi *et al*., 2023) are based on two-dimensional (2D) light images or the projected silhouettes from a batch of seeds, and can separate and process individual seeds. However, the analysis of the internal seed structures using different light microscopy modes requires seed dissection and usually produces 2D images. Several computer vision software tools and algorithms allow further recognition and segmentation of raw digital data in three-dimensional (3D) format. However, efficient capture of the entire 3D structure is difficult to achieve in this context, especially with relatively high sample throughput (Liu *et al*., 2024).

Replacing light with radiation outside the visible spectrum or using a wide region of the electromagnetic spectrum offers more opportunities for improving imaging power (Mooney *et al*., 2012; Fiorani and Schurr, 2013; Borisjuk *et al*., 2023). Hyperspectral imaging has recently gained attention for seed quality and safety inspection (Feng *et al*., 2019). However, only two techniques can aid in efficient non-invasive analysis of the internal structure of crop seeds: X-ray computed tomography (CT) and magnetic resonance imaging (MRI) (Stuppy *et al*., 2003; Van As *et al*., 2009; Borisjuk *et al*., 2013). Though both these modalities can generate 3D datasets non-invasively, they vary in terms of their working principles, strengths, and challenges. CT screening of seeds is relatively fast, with the availability of protocols for apparently high sample throughput. CT has been successfully used in plant science (Shi *et al*., 2022), including morphometric analysis of grains of wheat (Strange *et al*., 2015; Le *et al*., 2019; Xiong *et al*., 2019; Liu *et al*., 2020; Zhou *et al*., 2021), rice (Hu *et al*., 2020), maize (Hou *et al*., 2019), tomato (Gargiulo *et al*., 2020), soybean, and various nuts (Liu *et al*., 2020). CT is suitable for seed screening owing to its high spatial resolution and image quality. However, the technique has a few limitations, such as the use of ionizing radiation and the inability to differentiate among soft tissues (based solely on native contrast). Thus, tissue fixation and/or the use of contrast agents is often required to achieve the desired resolution (Wang *et al*., 2017; Duncan *et al*., 2022).

By using magnetic fields and radio waves, nuclear magnetic resonance (NMR) platform offers an alternative solution that is safe for both the seed and the researcher. Various NMR techniques are applicable in plant science including time domain NMR (TD-NMR) (Colnago *et al*., 2021; Windt *et al*., 2021) and various magnetic resonance imaging (MRI) approaches (Jahnke *et al*., 2009; Borisjuk *et al*., 2012, 2023; Van As and Van Duynhoven, 2013; Blystone *et al*., 2024). MRI has been applied not only for structural seed imaging but also for visualizing tissue-specific distribution of lipids, water, and metabolites in living seeds with reasonable spectral and spatial resolution (Radchuk *et al*., 2021, 2023; Rolletschek *et al*., 2021). Although MRI is more suitable than X-ray CT for studying the internal characteristics of seeds *in vivo*, it has some limitations, such as a lack of automated data analysis and low throughput. For instance, seed tissues/organs have to be manually segmented to calculate the volumes of the various components arising from the issue of automating this process. This is where deep learning (DL) approaches come in, the application of which has significantly boosted progress in image analysis in various scientific fields, including medicine, biology, and agriculture (Greenspan *et al*., 2016; Litjens *et al*., 2017; Pound *et al.*, 2017; Skolnick *et al*., 2017; Kamilaris and Prenafeta-Boldú, 2018; Toda *et al*., 2020; Selzner *et al*., 2023). Computer vision algorithms and the application of DL can be extremely useful in solving the issues in MRI data analysis. Plant MRI is a relatively new discipline and, unfortunately, has not produced enough public data to facilitate the learning process. However, this hindrance does not justify delaying the application of modern artificial intelligence techniques for image processing in plant MRI.

Cereals are the most important staple crops, and wheat alone accounts for ~20% of the calories and protein in the human diet worldwide (Hawkesford *et al*., 2013). The storage products are unevenly distributed among the various compartments of the grains of these crops. The starchy endosperm, which accounts for the bulk of the grain, contains the majority of the starch and is the source of white flour, whereas lipids and proteins are primarily deposited in the embryo and aleurone (a thin tissue layer surrounding the surface of the starchy endosperm). An elevated endosperm-to-embryo ratio, reduced seed coat thickness, and altered metabolic composition have helped obtain high-yielding crop grains in the course of domestication (Golan *et al*., 2015).

The primary idea of the study was to facilitate plant MRI image analysis by DL with the aim to improve performance of MRI-based phenotyping of wheat (*Triticum aestivum*) and barley (*Hordeum vulgare*) grains. This was achieved by (i) developing DL approaches by overcoming the limited availability of training data, (ii) prototyping data processing workflow, and (iii) testing the feasibility of this approach for relevant crops. The MRI-Seed-Wizard tool developed in this study revealed previously inaccessible traits in the cereal grains in a rapid and automated manner.

## Materials and methods

### Seed material

Representative mature grains of wheat and barley were used for both MRI and TD-NMR. The grains of wheat varieties Flair, Nirvana, Prinz, Toras, Welford, and Nadobna were received from IPK Genbank. The grains of founder lines of the National Institute for Applied Botany (NIAB) Diverse Multiparent Advanced Generation Intercross (MAGIC) population (called hereafter the NDM population) were kindly provided by Dr R. Sharma from Scotland’s Rural College (UK). The seeds of other material were produced as described earlier (barley: Radchuk *et al*., 2006, 2021; maize: Langer *et al*., 2023; rapeseed: Rolletschek *et al*., 2021).

### MRI measurements

High resolution 3D MRI of seeds was conducted at 9.4 T field strength using an Avance III HD 400 MHz NMR spectrometer (Bruker BioSpin, Germany) equipped with a ^1^H cryo probe head with inner diameter of 10 mm. For measurements, seeds were placed inside a thin glass walled tube with inner diameter of 5 mm or 10 mm (NMR Economy Sample Tube, SP Wilmad-Labglass, USA). The images were acquired by using a spin-echo sequence with global excitation and refocusing pulses, as described earlier (Munz *et al.*, 2016). The echo time (TE) was always set to be as short as possible for minimum T2 relaxation and maximum signal, whereas imaging conditions were adjusted experimentally. In particular, for measurements of barley and wheat specimens, the field of view (FOV) was set to 20 × 4.5 × 4.5 mm with a matrix size of 250 × 56 × 56 mm. Signal-to-noise ratio (SNR) was improved by increasing the number of averages where necessary. The isotropic resolution was increased by zero-filling in post-processing (e.g. from 80 µm to 40 μm). Application of the 11.7 T Avance Neo 500 MHz Super Wide Bore NMR spectrometer (Bruker BioSpin) equipped with a ^1^H quadrature probe head with inner diameter of 66 mm was demonstrated for multi-seed measurements. Details of corresponding MRI settings are provided in Supplementary Table S1.

Imaging of maize (*Zea mays*) and oilseed rape (*Brassica napus*) seeds was conducted inside of the custom-made holder, which was printed on a Form 2 3D printer (Formlabs, USA) using high temperature non-magnetic resin (type flhtam01 and flhtam02) ensuring 50 μm resolution printing. The 3D CAD program SolidWorks Standard 2019, version 27.0 (SOLIDWORKS Corp., USA) was used to create the 3D insert.

### Image pre-processing

Manual image segmentation was performed using Amira software (FEI Visualization Sciences Group, France). Most of the obtained images had nearly isotropic resolution of 40 × 40.01 × 40.01 μm per voxel (0.0400000 × 0.0401786 × 0.0401786 mm). For all samples the uniform image size 500 × 180 × 180 was adopted, using zero padding when required. For image processing, files were saved in Nifti format (Larobina and Murino, 2014).

### Software and frameworks for automatic semantic segmentation

The Python environment was used for development of the MRI segmentation pipeline and data post-processing while the PyTorch framework was applied for DL. A simplified version of U-Net (Ronneberger *et al*., 2015) was applied as the neural network type for the 2D segmentation model. Our U-Net version had lower depth (three levels instead of the original five), and a constant number of channels (64) on each level. The hyperparameter settings for training were Adam optimizer (Kingma and Ba, 2015), cross-entropy loss, batch size=64, number of epochs per session=100. The segmentation model for automatic 3D segmentation was nnUnet (Isensee *et al*., 2021). The selection, amount, and intensity of data augmentation, input normalization schema, tiling of input sample (number of chunks, if the volume does not meet device memory requirements when processed as whole), batch size, and other configurable settings were determined automatically based on the data fingerprint. Training sessions last 1000 epochs with standard setting, taking 3 d or more depending on the hardware capabilities. Alignment of (digital) samples in a co-ordinate grid was facilitated by the ResNet network (He *et al*., 2016). Training of neural net models was performed on an NVIDIA A40 GPU. Python mathematical packages such as scikit-image (van der Walt *et al*., 2014) helped to retrieve morphological properties from processed data.

### Analysis of seed traits

Morphometrical traits were determined based on different modalities. MRI-Seed-Wizard with manual and automatic segmentation (as described in ‘Image pre-processing’ and ‘Software and frameworks for automatic semantic segmentation’) provided algorithms for morphometric data (Table 1). In addition, grain area, length and width were measured using Marvin seed analyser (GTA Sensorik, Germany) following Gegas *et al*. (2010). Compositional traits (starch, moisture, protein, and lipid content) were measured using a combination of TD-NMR (MQ60 instrument, Bruker, Germany) and near-infrared spectroscopy (NIRS, MPA instrument, Bruker, Germany) as detailed earlier (Fuchs *et al*., 2013; Rolletschek *et al*., 2015; Keil *et al*., 2023).

**Table 1.** Glossary of abbreviations and explanations of grain traits

Abbreviation	Meaning
Grain traits determined using MRI-Seed-Wizard	
* V*_m_	Volume of monolith (mm^3^): *V*_m_=*C*_m_*r*^3^, where *C*_m_ is count of voxels in all segmented classes and *r* is resolution of original MRI image (assuming isotropic)
* V*_e_	Volume of embryo (mm^3^): *V*_e_=*C*_e_*r*^3^, where *C*_e_ is count of voxels in segmented embryo class and *r* is resolution of original MRI image (assuming isotropic)
* V*_en_	Volume of starchy endosperm (mm^3^): *V*_en_=*C*_en_*r*^3^, where *C*_en_ is count of voxels in segmented endosperm class and *r* is resolution of original MRI image (assuming isotropic)
* V*_a_	Volume of aleurone (mm^3^): *V*_a_=*C*_a_*r*^3^, where *C*_a_ is count of voxels in segmented aleurone class and *r* is resolution of original MRI image (assuming isotropic)
$\;V_{m}^{c}$	Convex volume of monolith (mm^3^): $V_{m}^{c}=C_{m}^{c}r^{3}$, where $C_{m}^{c}$ is count of voxels in convex volume of monolith [calculated by scikit-image computational geometry algorithm (van der Walt *et al.*, 2014)] and *r* is resolution of original MRI image (assuming isotropic)
$\;V_{e}^{c}$	Convex volume of embryo (mm^3^):$\;V_{e}^{c}=C_{e}^{c}r^{3}$, where $C_{e}^{c}$ is count of voxels in convex volume of embryo [calculated by scikit-image computational geometry algorithm (van der Walt *et al.*, 2014)] and *r* is resolution of original MRI image (assuming isotropic)
* A*_m_	Surface area of monolith (mm^2^): *A*_m_=$C_{m}^{q}$*r*^2^, where $C_{m}^{q}$ is count of unit squares on the outer isosurface of merged segmented classes [calculated with the Marching Cubes algorithm (Lorensen and Cline, 1987) implemented in scikit-image library (van der Walt *et al.*, 2014)] and *r* is resolution of original MRI image (assuming isotropic)
* A*_e_	Surface area of embryo (mm^2^): *A*_e_=$C_{e}^{q}$*r*^2^, where $C_{e}^{q}$ is count of unit squares on the isosurface of segmented embryo [calculated with the Marching Cubes algorithm (Lorensen and Cline, 1987) implemented in scikit-image library (van der Walt *et al.*, 2014)] and *r* is resolution of original MRI image (assuming isotropic)
* L*_m_	Length of monolith ^*a*^ (mm)—the dimension of segmented instance along its longest axis after alignment
* W*_m_	Width of monolith^*a*^ (mm)—the dimension of segmented instance on the axis perpendicular to the longest axis and parallel to the virtual floor after alignment
* D*_m_	Depth of monolith ^*a*^ (mm)—the dimension of segmented instance on the axis perpendicular to the length and width axes after alignment
* D*_c_	Depth of crease ^b^ (mm)—the height of anatomical hollow fold on a segmented instance, measured in the middle slice after alignment
* L*_m_/*W*_m_	Ratio of monolith length to its width
* V*_e_/*V*_en_	Ratio of embryo volume to endosperm volume
* R*_a_	Volume ratio aleurone to monolith: *R*_a_=*V*_a_/*V*_m_
* R*_e_	Volume ratio embryo to monolith: *R*_e_=*V*_e_/*V*_m_
* R*_en_	Volume ratio endosperm to monolith: *R*_en_=*V*_en_/*V*_m_
* S*_m_	Sphericity of monolith: surface area of a sphere of the same volume as the monolith divided by the actual surface area of the monolith: $S_{m}=\frac{\pi^{\frac{1}{3}}{\left(6V_{m}\right)}^{\frac{2}{3}}}{A_{m}}$
* S*_e_	Sphericity of embryo: $\;S_{e}=\frac{\pi^{\frac{1}{\text{3}}}{\left(6V_{e}\right)}^{\frac{2}{\text{3}}}}{A_{e}}$
* T*_m_	Solidity of monolith (‘convexity factor/ratio/index’): *T*_m_**=***V*_m_/$V_{m}^{C}$ Solidity measurement of the overall concavity of an object (‘geometric density’). It represents the amount of space that is filled within the convex hull of the object
* T*_e_	Solidity of embryo (‘convexity factor/ratio/index’): *T*_e_=*V*_e_/$V_{e}^{C}$
SEF	Starch enrichment factor—reflects the level of starch in endosperm: SEF=*G*_s_*×V*_en_
LEF	Lipid enrichment factor—reflects the level of lipid in embryo and aleurone: LEF*=G*_l_*×(V*_e_*+V*_a_)
Morphometric parameters additionally determined by MARVIN seed analyser	
Area	Average area of 2D profile of grain sample (mm^2^)
Length	Average length of grain sample (mm)
Width	Average width of grain sample (mm)
Compositional parameters additionally determined by TD-NMR and NIRS	
* G*_s_	Grain starch content (%)
* G*_m_	Grain moisture content (%)
* G*_p_	Grain protein content (%)
* G*_l_	Grain lipid content (%)

^*a*^ After Tanabata *et al.* (2012).

^*b*^ According to Strange *et al.* (2015).

### Basic outline of MRI-Seed-Wizard application

Prior to using the MRI-Seed-Wizard application, the MRI scanning conditions and the number of seeds for each measurement have to be optimized. The resolution should be chosen carefully, considering the characteristics of the available MRI scanner and ensuring it is sufficient for the desired morphometric assessment. After image acquisition, the 3D MRI data should be saved in Nifty format and then transferred to the segmentation model, which is based on nnUnet and requires a Python environment, preferably with GPU hardware. The resulting segmented data are further processed using a series of Python scripts provided here to extract and compute the morphometric features. Data from each processing step are saved for tracking if necessary. The final morphometric results in tabular format can be used for comparative and statistical analysis. More details and examples of the Wizard application are presented in the published codebase (https://github.com/akvilonBrown/mri-wizard).

### Statistical methods

Statistical processing was performed in MATLAB (MathWorks, Natick, MA, USA). For statistical processing of obtained results, the method of power calculation was applied as described by Cohan (1988). The method allows estimating required samples size for future investigations, with a certain level of confidence and for various volumetric and morphometric properties. The corrcoef function (Matlab R2020b), used for calculation of *P-*values in correlation analysis of traits, is computed by transforming the correlation to create a *t*-statistic having *N*−2 degrees of freedom where *N* is the number of rows of the data matrix. Normality tests (Ghasemi and Zahediasl, 2012) were applied to check a normal distribution of the data. A threshold for *P-*values of 0.01 was considered as sufficient to reject assumption of normality. The visual evaluation included quantile–quantile (QQ) plots (Marden, 2004).

## Results

### Creation of an initial (pilot) model for seed segmentation based on limited MRI datasets

**Fig. 1.**
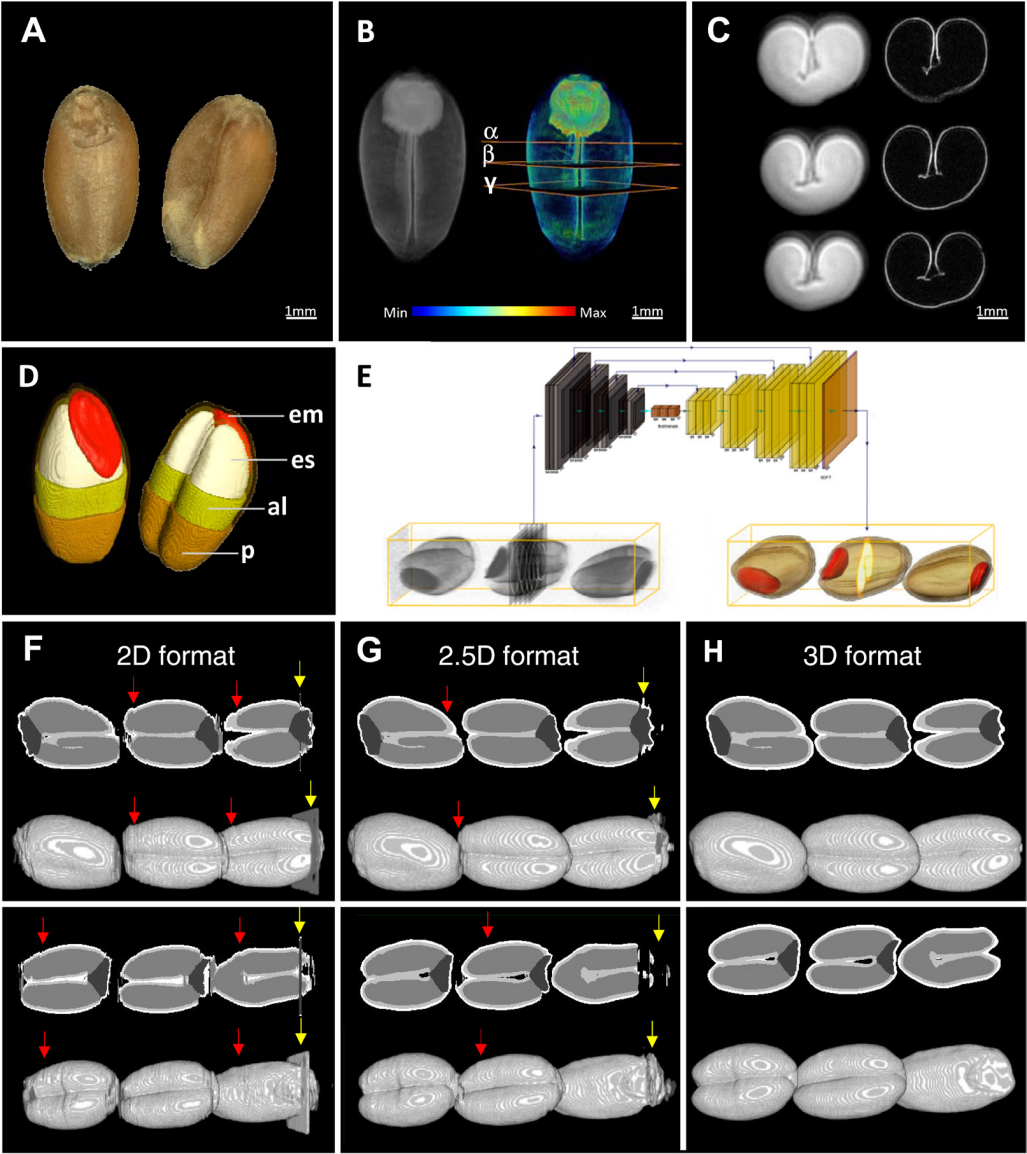
Magnetic resonance imaging (MRI) and processing of individual mature wheat grains. (A) Optical images of the mature wheat grains. (B) 3D visualization of a grain by ^1^H-MRI (left) and lipid localization in the same grain by MRI (right). Lipid levels are color-coded. Positions for the virtual cross-sections shown in (C) are labeled by α, β, and γ. (C) Virtual cross-sections of a grain showing the endosperm (bright signal, left) and aleurone layer (right). (D) MRI-based manual segmentation of the main organs of a wheat grain, showing the internal structures. (E) MRI processing by 2.5D convolutional neural network (U-Net). A stack of virtual cross-sections from the source was taken as input to produce a single segmented cross-section. The volumetric context of adjacent slices was considered to a certain depth (10 slices in both directions from the targeted slice), eventually producing smoother surfaces on the segmented objects reconstructed from the inferred slices. (F–H) Segmentation results from two independent measurements (upper and lower panels) of grains from models processed in 2D, 2.5D, and 3D formats. (F) 2D segmentation with independent processing of slices resulted in an inconsistent surface after reassembling into a single object (red arrows). Some images contain faulty frames with spurious noise, which is misinterpreted by the 2D model (yellow arrows). (G) 2.5D segmentation appeared more robust to fluctuations between slices. While the contextual information from the adjacent slices helps maintain smoother surfaces, peripheral slices still underperformed (red arrows). Misinterpretation of faulty frames was not fully suppressed (yellow arrows). (H) 3D segmentations genuinely processed the volumes in all dimensions, eventually producing holistic shapes. Bar length: 1 mm. Abbreviations: al, aleurone; em, embryo; es, endosperm; p, pericarp.

MRI was applied to visualize seed structure and lipid distribution (as a marker for aleurone and embryo) within mature grains of wheat (Fig. 1A–D). We generated 3D models of grains representing six distinct wheat cultivars (Supplementary Fig. S1). The main grain compartments were estimated by manually segmenting individual grains (Supplementary Table S2). The initial number of 3D MRI and manually segmented images was insufficient to start with supervised segmentation, which usually requires much larger source and target data. Next, we attempted to cope with the limited 3D data.

First, the 3D images of each grain were manually segmented into four target tissues: embryo, starchy endosperm, aleurone, and pericarp, which are the main compartments in cereal grains (Fig. 1D). These tissues were assigned to enumerated classes (a total of five, including background). The segmented and source 3D MRI images of each seed were virtually cut to produce 2D sections. One 3D nuclear magnetic resonance grain image with dimensions of 500 × 180 × 180 voxels provided approximately 400 cross-sections of seed samples equally distributed along the longest axis. These 2D data were then used for training the pilot model. Since 2D models are less demanding in terms of data, even a few (five to seven) initial samples are sufficient to begin training a pilot model that kickstarts the iterative process of learning. In our dataset, though the processing of volumetric images in 2D did not take all dimensions into account, it helped generate a sufficient number of images to begin the learning process.

To further increase the number of 2D images, each 3D specimen was virtually rotated at small angles (5–20° to remain within predefined dimensions), thus introducing more variations in 2D sections. A total of 53 MRI and 53 segmented images (53 pairs) were generated from the initial dataset. Among them, 36, 9, and 8 pairs were assigned to the training, validation (which helps measure performance during the course of training), and test data (which helps check the performance after training), respectively. The training, validation, and test data had no overlaps and comprised 9113, 2265, and 2093 images, respectively, in 2D, with a size of 180 × 180 pixels for each image. Thus, sufficient training data were obtained for the pilot model.

This initial dataset was utilized for the training segmentation model using a simplified version of U-Net (Ronneberger *et al*., 2015), which has previously been successfully tested for the segmentation of biomedical images (Fishman *et al*., 2021). Then, we evaluated model performance using standard metrics, such as the Dice score (Sorensen–Dice score or F1 score; Dice, 1945; Sorensen, 1948). The average Dice score of four classes (1–4), ignoring background, after training the pilot model was 0.851. Though this score was still insufficient (as the new segmented samples would require expert corrections), it already led to time savings by an order of magnitude.

### Enhanced model performance after moving to frame stack processing

The pilot model helped determine optimal hyperparameters and on-the-fly data augmentation for subsequent training sessions. Empirically, with extensive hyperparameter grid search we defined the optimal values that have the best impact on the model training and its evaluation on the test set. For the data scaling scheme, we picked standardization on the volume level, which involved subtracting the mean signal intensity from each voxel value and dividing the result by the standard deviation. The optimal batch size, which is the number of input 2D images for a single forward pass through the neural network, was found to be 64. We used Adam (Kingma and Ba, 2015) as the popular neural network optimizer and categorical cross-entropy as the loss function, typical for multi-class segmentation tasks. On-the-fly augmentations that appeared effective in the empirical search of the best combination included random flips, rotations, scaling, shifting, random brightness and contrast perturbation, Gaussian noise, and a custom noise imitating faulty frames that randomly appear on some images. The training session had a fixed number of traversals through all images in the training set (epoch). We set the maximum number of 100 epochs for all training sessions and picked the model with the least validation error. Usually, convergence was reached within the 40th–50th epoch for the pilot and subsequent models. However, assembling the inferred sections into a 3D stack revealed a coarse surface and unstable predictions at the periphery of a segmented grain, which was a limitation of the model. This inaccuracy was attributed to the independent processing of samples in a 2D format, which compromised the quality of 3D reconstruction (Fig. 1F; Supplementary Video S1).

To enhance the performance of the pilot model, we simultaneously processed multiple slices in a stack (Fig. 1E). This approach introduced dependencies among the frames in the third dimension, leading to improved segmentation accuracy and smoother surfaces on the resulting objects. Convolution layers are designed to receive multiple frames as input, making the implementation straightforward. This technique is sometimes referred to as a 2.5D format, as it helps obtain context in the third dimension while conceptually remaining a 2D segmentation process (Han, 2017; Wardhana *et al*., 2021). In our pipeline, several sections from the source MRI scan were processed as a stack, considering the volumetric context of adjacent slices up to a depth of 10 slices in both directions. This stack corresponded to a single annotated target, which was a segmented version of the central slice in the input (Fig. 1E). The stack moved like a sliding window, and the model established correspondence with the next single annotated target. This method improved the segmentation accuracy compared with the ordinary 2D approach and produced smoother surfaces on the resulting segmented objects (Fig. 1G; Supplementary Video S1).

### Transition to 3D format as a key step toward automated segmentation

**Fig. 2.**
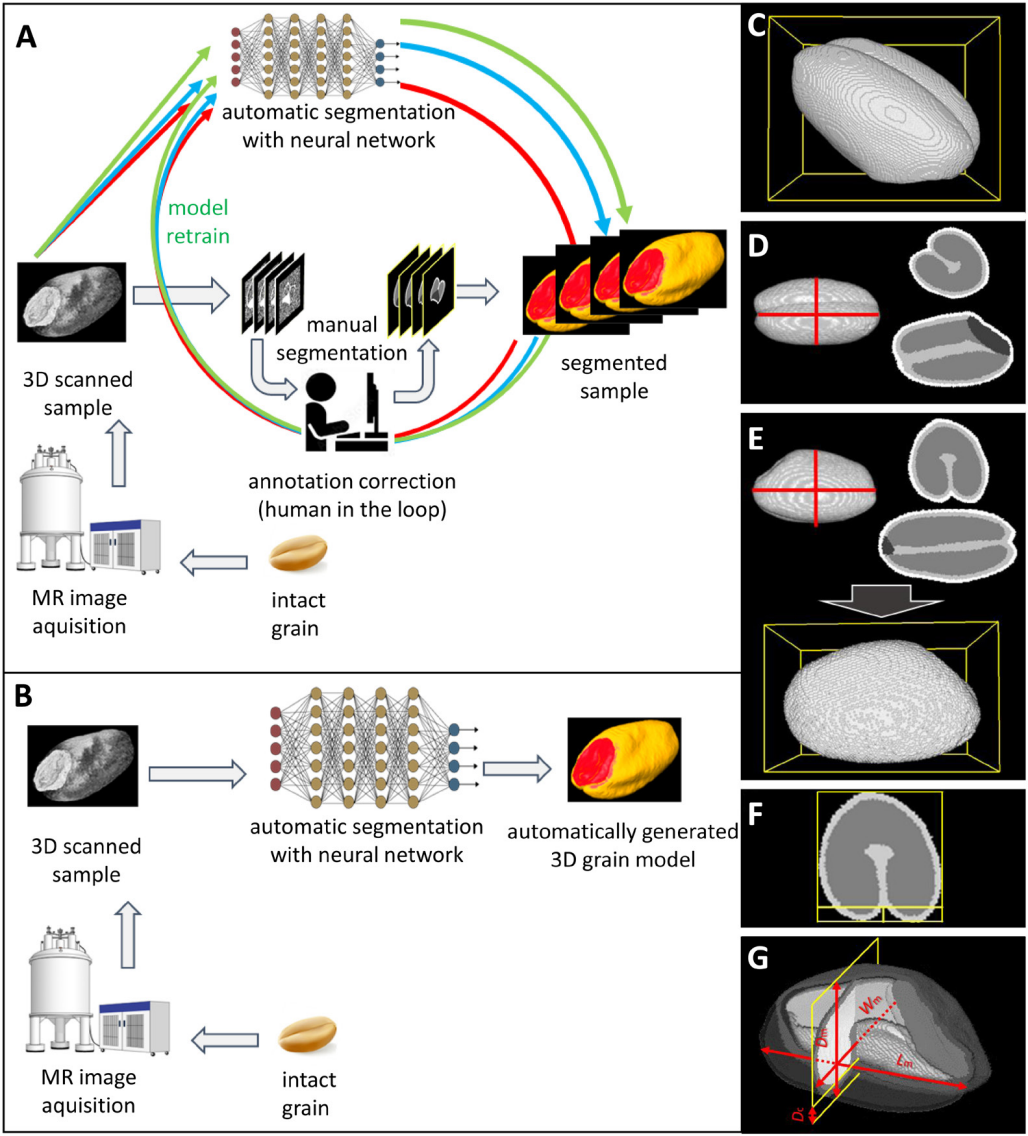
Iterative learning pipeline with the fully supervised approach that utilizes source–target pairs for training a model and post-processing of a segmented and separated wheat grain. (A) The first annotated samples sufficient to begin the training of the pilot model were produced by a human expert. In the early stages of model development, the human expert corrected the predictions made by the pilot model to enrich the training set for the next training iteration—‘human in the loop’. (B) After obtaining a larger dataset, the model was replaced by a 3D variant and the source data were treated as 3D objects producing high-quality segmentation results without human assistance. (C) A general view of a segmented and separated grain. (D) Only the alignment along the length of a grain was achieved using principal component analysis. The width and depth of a grain and crease were not guaranteed to match intuition. Red lines indicate the cross-sections shown on the right. (E) The final alignment, performed using ResNet, placed objects in a uniform position for all grains. (F) This position of the grains also simplified the measurements of the crease depth on a central slice. (G) Measurement of some dimensions and crease depth in an aligned instance. Abbreviations: *D*_c_, depth of crease; *D*_m_, depth of monolith; *L*_m_, length of monolith; *W*_m_, width of monolith.

We brought into the analysis the MRI scans of the grains of NDM founder lines (Scott *et al*., 2021). The images were then processed by the model, corrected by a human expert, and added to the training set for subsequent training iterations (Fig. 2A). This approach significantly increased the prediction accuracy with each iteration, reaching the test Dice score of 0.884. After the addition of 10 new MRI scans, the score increased to 0.892 and began to reach the upper limit of the model’s potential (the addition of the 16th image increased the Dice score to only 0.893).

After the aggregation of sufficient data with a 2.5D segmentation pipeline, a 3D self-configuring segmentation framework nnUnet (Isensee *et al*., 2021) was applied to automatically process the next batch of 3D MRI scans. We trained nnUnet on all available data from the grains of the six initial cultivars and 16 NDM founders produced during the gradual improvement of the 2.5D model. The framework achieved high performance with a mean Dice score of 0.904 on test data. The visual appearance of these segmented samples proved to be more accurate than the 2D and 2.5D segmentation models (Fig. 1H; Supplementary Video S1). Predictions from the new models required little or no correction, with no significant impact on the volumetric properties of the tissues. Using this approach, we performed automated segmentation of the MRI scans of 96 representative grains with positive feedback during human inspection of the results (Fig. 2B). Thus, we were able to obtain a high-performance automatic process to analyse grain morphometry: the 3D nnUnet segmentation framework became the main segmentation tool, while 2D and 2.5D segmentation models were used as intermediate steps to expedite the progress in the conditions of scarcity of data on initial stages.

### Automated segmentation efficiently facilitated morphometric analysis

To acquire the morphometric data from the segmented grains, the 3D images were separated into individual objects (instances) (Fig. 2C) using classical computer vision algorithms, such as clustering and watershed segmentation (Beucher and Lantuejoul, 1979). The embryo voxels served as a basis for clustering, and the centers of these clusters became starting points for inflating instance labels outwards to the surface between non-background and background voxels using a distance map. The instances from the segmented data were centered and saved in a Nifty file with a size of 200 × 180 × 180 voxels. Simultaneously, we saved the source instances using annotated instance data to carve out relevant volumes from the source files.

A uniform placement of the grains in space was achieved in two steps. First, we exploited the fact that grains have elongated shapes and applied an algorithm, commonly used in principal component analysis (PCA), to align the digital objects of grain in an orthogonal 3D MRI grid relative to the longest axis of the grain (Fig. 2D). This algorithm treats coordinates of segmented voxels as data points and is able to determine principal axes to reorient the object by laying the grain horizontally in a volume grid. However, the orientation of the grain crease was not always accurate (for example, the width was not always greater than the depth), thereby limiting the application of the PCA-related technique. We then used a neural model ResNet (He *et al*., 2016) in regression format to infer the final angle for the adjustment of instance orientation. This model helped predict the rotation angle of the crease from the desired bottom position on the central planar slice of the centered grain. We set two target values, the cosine and sine of the angle, to make the model behave smoothly on the value transition between −180 and +179°. With the angle value of the final roll, it became possible to combine two rotations and align objects uniformly (Fig. 2E).

At each post-processing step, we used Python scripts and additional open-source packages to assess the basic morphometric traits of the grain. The volumes of each tissue and the whole grain were calculated as the number of voxels that make up the respective tissue multiplied by the unit volume of each voxel derived from the image resolution. We also calculated the surface area for the whole grain and embryo using the Marching Cubes algorithm (Lorensen and Cline, 1987) implemented in the scikit-image library (van der Walt *et al*., 2014). We retrieved these traits directly after instance separation since they did not depend on the orientation of the objects. The other traits (grain length, depth, width, and crease depth) were confidently retrieved after ResNet-aided alignment. The crease depth was calculated by measuring the distance between the bottom of an object in the central section and the farthest point of the crease profile (Fig. 2F–G). The crease profile was defined as the difference between the initial object shape and the convex shape on the central section.

We compared a number of extracted morphological features, retrieved by MRI-Seed-Wizard, with manually measured wheat grain dimensions, conducted with a sliding caliper (accuracy 0.05 mm). The MRI-Seed-Wizard output showed high correlation with the mechanical measurements of wheat grain dimensions (Supplementary Table S3; Supplementary Fig. S2). The difference plot, constructed according to Bland and Altman (1986), demonstrated that biases for each dimension were not significant (also considering that MRI-Seed-Wizard output was based on 0.08 mm initial scanning resolution and the accuracy of mechanical measurements was 0.05 mm), since the zero difference is within a confidence interval (95%) of the mean of differences.

The variations of differences were random and showed the limits of agreement (calculated as mean ±1.96 SD according to the Bland–Altman method) from −0.45 to 0.36 mm for length, from −0.14 to 0.16 mm for width, and from −0.17 to 0.2 mm for depth. Relative differences with respect to the mechanical measurement were of comparable extent for all dimensions (Supplementary Fig. S3).

In spite of the limited number of manual measurements (48), the comparison with the MRI-Seed-Wizard calculations show a trend towards consistency. Presumably, the accuracy of segmentation and respective dimensional traits will be increased with higher resolution of the model. In a high-throughput scenario, the random nature of deviations should be mitigated by a high number of samples. With the potential spread of the application of MRI in the plant domain, a closer analysis would help to further improve the MRI-Seed-Wizard tool.

The developed segmentation process backed by DL and the post-processing pipeline formed the skeleton of the newly proposed MRI-Seed-Wizard tool. The first stage of MRI-Seed-Wizard ensured accurate high-throughput automated segmentation of seed MRI. The post-processing part of MRI-Seed-Wizard was responsible for arranging the segmented data and calculating the morphometric properties. The most important properties of grains derived from MRI-Seed-Wizard are listed in Table 1.

### Application of MRI-Seed-Wizard for comprehensive analysis of wheat seeds

**Fig. 3.**
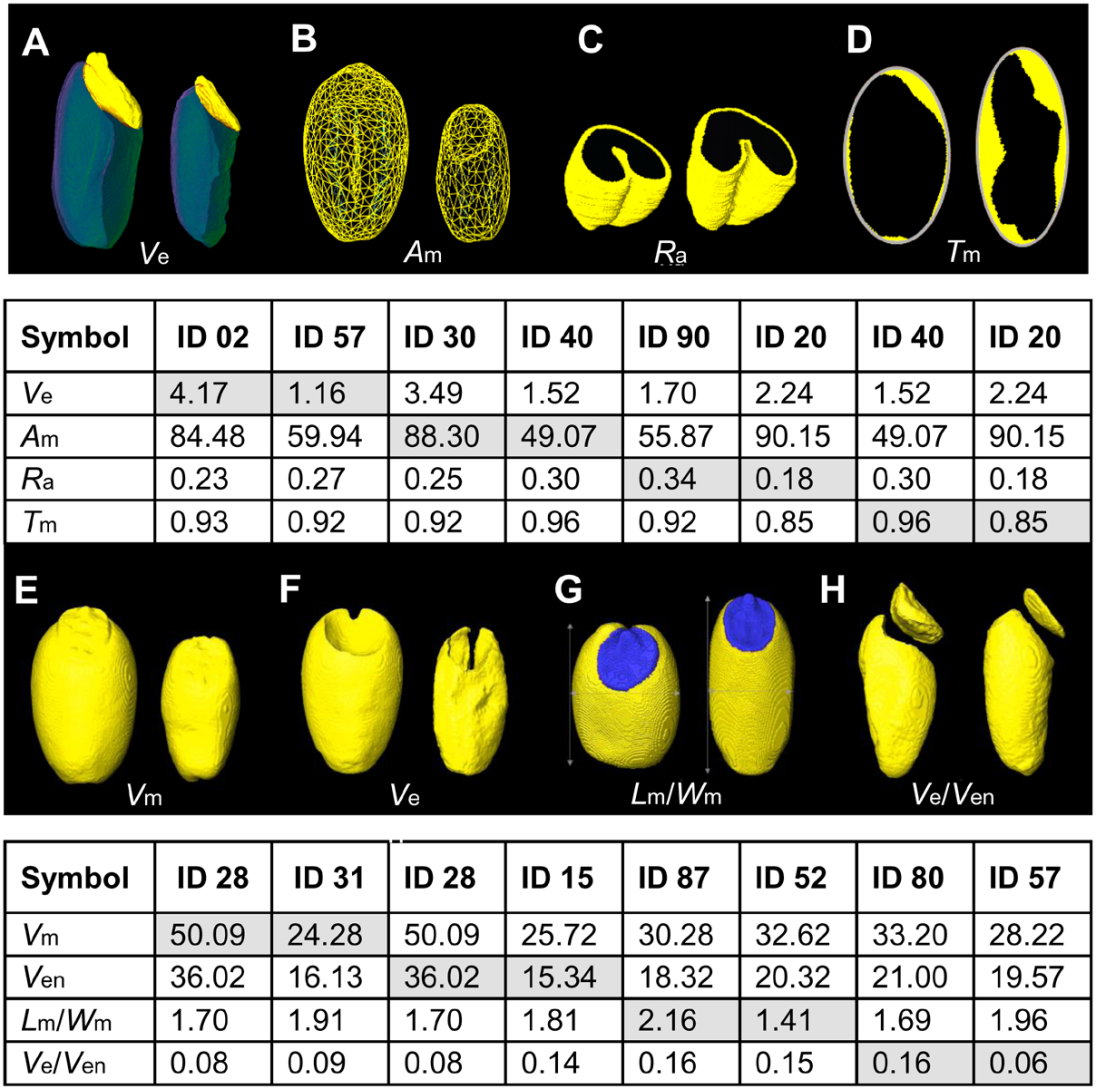
Assessment of the most contrasting single-grain morphometrical parameters using MRI-Seed-Wizard. (A) Biggest versus smallest embryo (cv. Banco versus Maris Fundin). (B) Largest versus smallest surface area of the monolith (cv. Cordiale versus Gladiator). (C) Largest versus smallest aleurone (cv. Stetson versus Copain). (D) Highest versus lowest solidity of the monolith (cv. Gladiator versus Copain). (E) Biggest versus smallest monolith volume (cv. Cordiale versus Flamingo). (F) Biggest versus smallest endosperm volume (cv. Cordiale versus Brigadier). (G) Highest versus lowest ratio of monolith length to width (cv. Steadfast versus Kloka). (H) Highest versus lowest ratios of embryo-to-endosperm volumes (cv. Spark versus Maris Fundin). IDs of wheat genotypes are provided in Supplementary Table S4. *A*_m_, surface area of monolith; *L*_m_/*W*_m_, ratio of monolith length to its width; *R*_a_, volume ratio aleurone to monolith; *T*_m_, solidity of monolith; *V*_e_, volume of embryo; *V*_e_/*V*_en_, ratio of embryo volume to endosperm volume; *V*_m_, volume of monolith; *V*_en_, volume of starchy endosperm.

We analysed mature grains of 16 NDM founder lines of the MAGIC wheat collection (Scott *et al*., 2021) using MRI-Seed-Wizard to attain novel insights into their trait interrelations. First, we illustrated some morphometric traits (not accessible by optical tools) in individual grains with contrasting phenotypes (Fig. 3). Cultivar (cv.) Gladiator (ID 40) seeds exhibited the highest solidity (*S*_e_) and smallest surface area of the monolith (*A*_m_), cv. Cordiale (ID 28) seeds exhibited the biggest monolith (*V*_m_) and endosperm volumes (*V*_en_) (endosperm is the major contributor of the monolith volume), cv. Maris Fundin (ID 57) seeds had the smallest embryo volume (*V*_e_) and embryo-to-endosperm ratio, and cv. Copain (ID 20) seeds harbored the lowest aleurone fraction and *S*_e_ (Fig. 3). This finding suggested that aleurone thickness might be more closely associated with the surface area of monolith.

Overall, 30 traits based on MRI-Seed-Wizard and parallel measurements using TD-NMR, NIRS, and MARVIN were collected (Supplementary Table S4). These data were used as a basis for comprehensive statistical analysis with the statistical power (Cohen, 1988) between 0.96 and 0.99 for most traits (Supplementary Table S5). The majority of the traits were assumed to have a normal distribution (Supplementary Fig. S4; Supplementary Table S5).

**Fig. 4.**
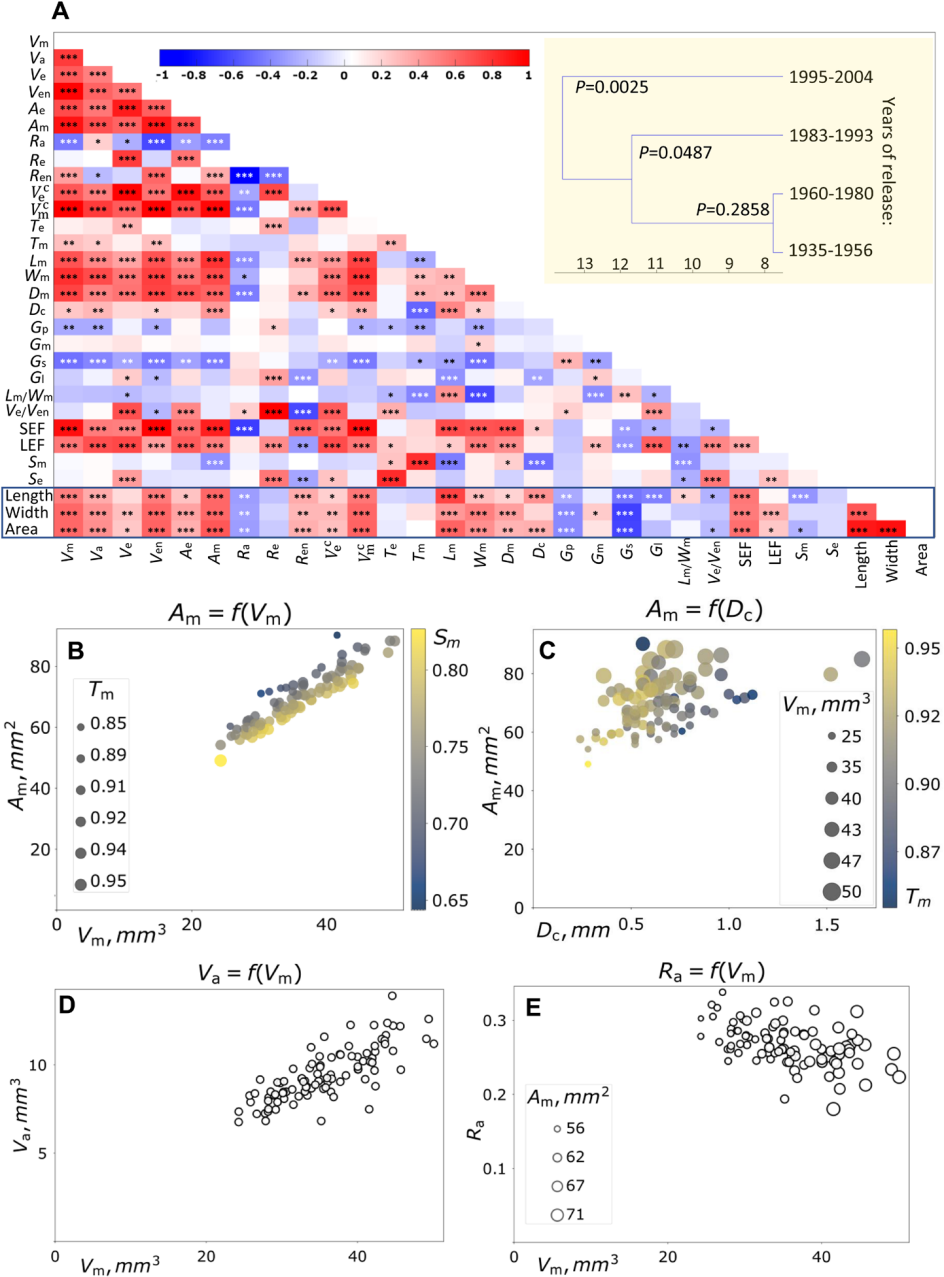
Correlation analysis of wheat grain traits. (A) Correlation plots of the phenotypes of grains belonging to the founders of the wheat NDM MAGIC population. The plots were constructed using data from MRI-Seed-Wizard. Red and blue colors indicate positive and negative correlation, respectively. The darker the color, the greater the absolute value of correlation. *P<0.05, **P<0.01, and ***P<0.001 as determined using the corrcoef command of Matlab (R2020b). Abbreviations are delineated in Table 1. The indicated framed features were acquired from the MARVIN analyser. Yellow inlet shows a hierarchical cluster dendrogram of the traits of 16 founders grouped by the decade of release based on complete distance linkage. (B) The correlation of the surface area shown as a function of the volume of the monolith. The solidity of monolith is encoded by the size of dots, and the sphericity is encoded by their color. (C) Correlation of the surface area of monolith shown as a function of the crease depth. The monolith volume is encoded by the size of dots, and the solidity is encoded by their color. (D) The correlation of the aleurone volume shown as a function of the monolith volume (no further traits encoded, size and color of the dots are uniform). (E) Correlation of the fraction of aleurone shown as a function of the monolith volume. The surface area of the monolith is encoded by the size of dots. *A*_m_, surface area of monolith; *D*_c_, depth of crease; *D*_m_, depth of monolith; *G*_l_, grain lipid content; *G*_m_, grain moisture content; *G*_p_, grain protein content; *G*_s_, grain starch content; LEF, lipid enrichment factor; *L*_m_, length of monolith; *L*_m_/*W*_m_, ratio of monolith length to its width; *R*_a_, volume ratio aleurone to monolith; *R*_e_, volume ratio embryo to monolith; *R*_en_, volume ratio endosperm to monolith; *S*_e_, sphericity of embryo; SEF, starch enrichment factor; *S*_m_, sphericity of monolith; *T*_e_, solidity of embryo; *T*_m_, solidity of monolith; *V*_a_, volume of aleurone; $V_{e}^{c}$, convex volume of embryo; $V_{m}^{c}$, convex volume of monolith; *V*_e_, volume of embryo; *V*_e_/*V*_en_, ratio of embryo volume to endosperm volume; *V*_m_, volume of monolith; *V*_en_, volume of starchy endosperm; *W*_m_, width of monolith.

Several morphometric features processed by MRI-Seed-Wizard were directly or indirectly related to the monolith volume as a proxy trait to grain size (Fig. 4A). We observed a linear correlation between the surface area of monolith (*A*_m_) and the volume of monolith (*V*_m_) (*r*=0.92, *P*<0.001). (Fig. 4B). In theory, surface area should increase more slowly because this quadratic function of area is slower than the cubic function of volume. Thus, the linear dependency in our observations indicated an interplay of other factors and the insufficiency of the range of volumes to showcase the theoretical assumptions. The variation within this dependency revealed the impact of solidity (*T*_m_) and sphericity (*S*_m_) (Fig. 4B). Seeds with higher *S*_e_ and *S*_m_ had smaller surface area. The depth of the crease (*D*_c_) was another major factor influencing the surface area (*A*_m_) and, consequently, solidity (Fig. 4C). Samples with similar crease depth but bigger monolith volume had larger surface area.

Furthermore, aleurone volume (*V*_a_) positively correlated with volume of monolith (*V*_m_) (*r*=0.77, *P*<0.001) (Fig. 4D). However, the fraction of aleurone to monolith (*R*_a_) decreased with *V*_m_ (*r*=–0.44, *P*<0.001) (Fig. 4E). This dependency was impacted by surface area (*A*_m_) but not sphericity (*S*_m_). For instance, samples with larger surface area had larger fraction of aleurone. The highest negative correlation was found between the endosperm-to-monolith ratio (*R*_e_) and the aleurone-to-monolith ratio (*R*_a_) (*r*=–0.90, *P*<0.001). With increasing endosperm volume (*V*_en_), the fraction of the aleurone in monolith decreased (*r*=–0.62, *P*<0.001). While the area of monolith surface increases with the growth of monolith volume, the thickness of the aleurone and, therefore, volume of aleurone (*V*_a_) is not significantly impacted. The features of the aleurone layer per se would bring more clarity to the analysis and should be of interest in future research. The dependencies with aleurone volume (*V*_a_) are even more significant when considering the fact that aleurone and embryo are the main contributors to the oil content of seeds and aleurone fraction in monolith (*R*_a_)>embryo fraction (*R*_e_).

To highlight the relationship between grain structure (volumes and fractions of different tissues) and composition (starch, protein, water, and lipid content), we introduced two new derivative features: lipid enrichment factor (LEF) and starch enrichment factor (SEF). These factors take starch and lipid contents into account and help correlate them to the volume of tissues that accumulate them. We found that LEF significantly correlated with embryo volume (*V*_e_) (*r*=0.73, *P*<0.001) and SEF expectedly correlates with endosperm volume (*V*_en_) (*r*=0.98, *P*<0.001). However, we observed a weak negative correlation between grain starch content (*G*_s_) and several other traits, except grain protein content (*G*_p_) (*r*=0.32, *P*<0.01). We observed a significant negative correlation between *G*_s_ and dimensional features, such as monolith width (*W*_m_; *r*=−0.53, *P*<0.001) and monolith volume (*V*_m_; *r*=−0.51, *P*<0.001), indicating lower density of starch deposits in bigger seeds. In contrast, grain protein and moisture content (*G*_m_) only mildly correlated with the other traits.

To gain insights into the historical background of the selection among the wheat varieties, we divided all 16 cultivars into four groups, each group containing four cultivars based on their decade of release. PCA could only aid in recognizing a separate cluster of the oldest cultivars. Hierarchical clustering revealed that the group with the oldest cultivars had similar phenotypic grain parameters to the group with the second oldest cultivars (released between 1969 and 1980). The groups with younger cultivars (released in 1983–1993 and 1995–2004) separated into two groups (Fig. 4A, inlet). Based on the year of cultivar release, a negative correlation was observed among grain lipid content (*G*_l_) (*r*=−0.21, *P*<0.01), monolith length (*L*_m_) (*r*=−0.23, *P*<0.05), and whole grain length (*r*=−0.18, *P*<0.001), indicating that newer cultivars produced shorter grains with lower lipid content.

### Versatility of MRI-Seed-Wizard in analysing barley seeds

We next assessed the applicability of MRI-Seed-Wizard in analysing barley seeds. Despite the apparent similarity in the shape and tissue composition of barley and wheat grains (Supplementary Fig. S5), applying the wheat segmentation model directly to barley grains resulted in a poorer visual quality than wheat grains. Some samples were acceptable but still required moderate corrections by a human expert, while in a few cases, the seeds and background were misinterpreted. To address these limitations, we added six manually corrected segmented images of barley grains to the existing dataset of wheat samples and retrained the model. The updated version of the model exhibited significantly improved segmentation quality for barley grains while maintaining the high quality for wheat grains.

The model was then applied to automatically segment and comparatively analyse the grains of the modern variety Barke, the old variety Golden Promise, and the transgenic lines with altered expression of genes involved in grain filling (Supplementary Table S6). The transgenic line N91 was characterized by transcriptional suppression of the *Jekyll* gene (Radchuk *et al*., 2006) while the lines VPE2i-11 and VPE2i-19 exhibited down-regulated *VPE2a–VPE2d* expression (Radchuk *et al*., 2021).

**Fig. 5.**
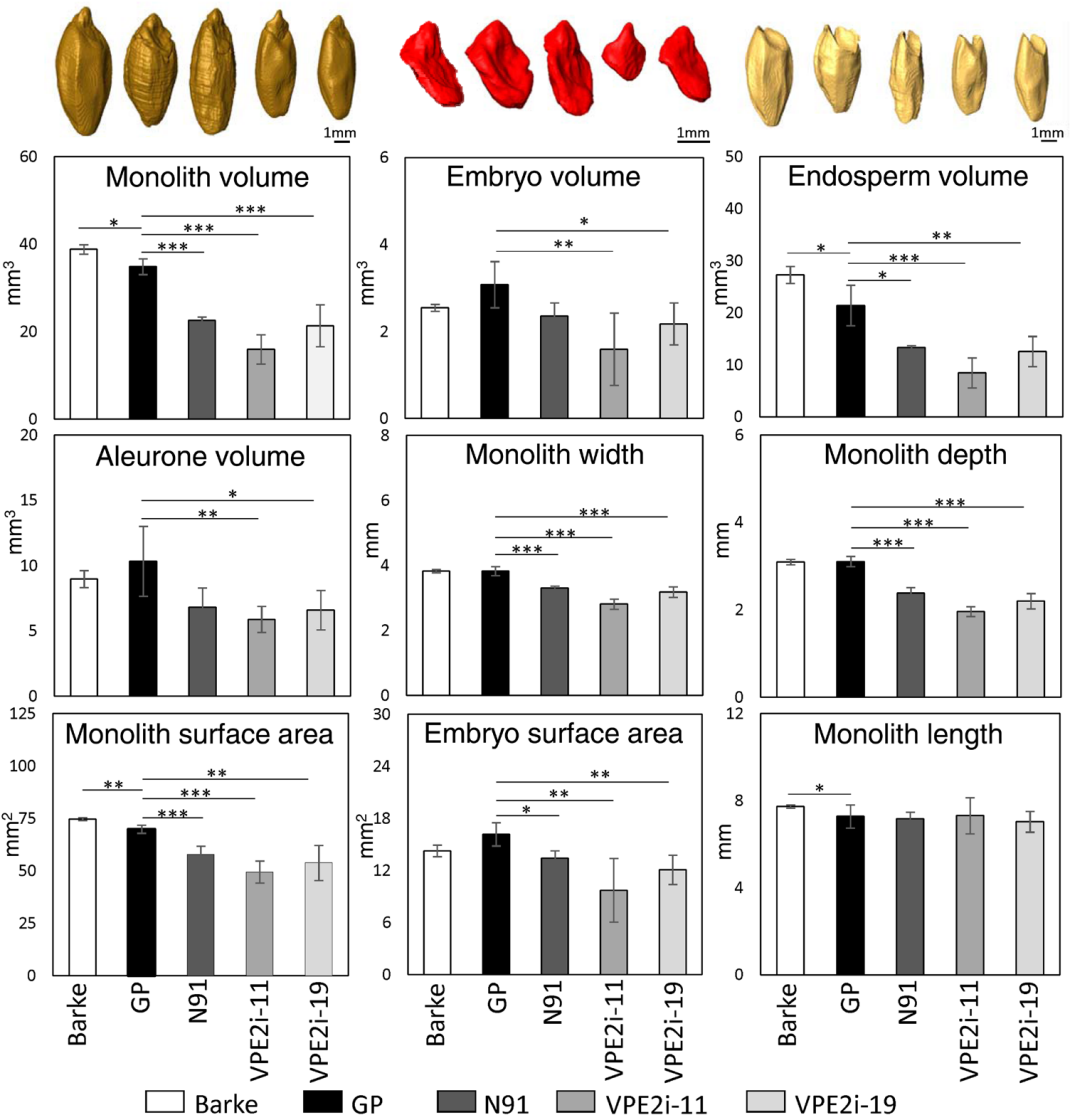
Phenotyping of wild-type and genetically modified barley grains using the MRI-Seed-Wizard tool. Magnetic resonance imaging (MRI) was used to detect the major phenotypic traits of Barke and Golden Promise (GP) cultivars as well as the transgenic grains with suppressed *Jekyll* (N91) and *VPE2* (VPE2i-11 and VPE2i-19) gene expression. The graphs in the upper panel showing the 3D shape and size of monolith, embryo, and endosperm in the respective genotypes (representative images). Values in the graphs represent means, and error bars represent standard deviation. *n*=3–6 biological replicates (individual caryopses), *P<0.05, **P<0.01, and ***P<0.001 as determined using the two-tailed Student’s *t*-test between GP and other accessions.

Compared with the Golden Promise grains (the starting cultivar for the transgenic lines), the VPE2i-11 and VPE2i-19 grains showed drastically lower monolith volume, surface area, starchy endosperm and aleurone volumes (*V*_m_, *A*_m_, *V*_en_, and *V*_a_), primarily owing to markedly lower monolith depth and width (Fig. 5). In addition, the transgenic seeds exhibited higher monolith length (*L*_m_) values. These findings were in accordance with the results of a previous study (Radchuk *et al*., 2021) that reported smaller endosperm and embryo volumes (*V*_e_ and *V*_en_) in the transgenic grains. Furthermore, we recorded lower *V*_en_ and *V*_a_ in N91 grains than in Golden Promise grains (Fig. 5), which might explain the previously described lower caryopses’ weight in this transgenic line (Radchuk *et al*., 2006). Overall, the MRI-Seed-Wizard tool could accurately analyse both wheat and barley grains, with the ability to detect the traits of genetically modified seeds.

### Multi-seed MRI substantially increased throughput needed for phenotyping

**Fig. 6.**
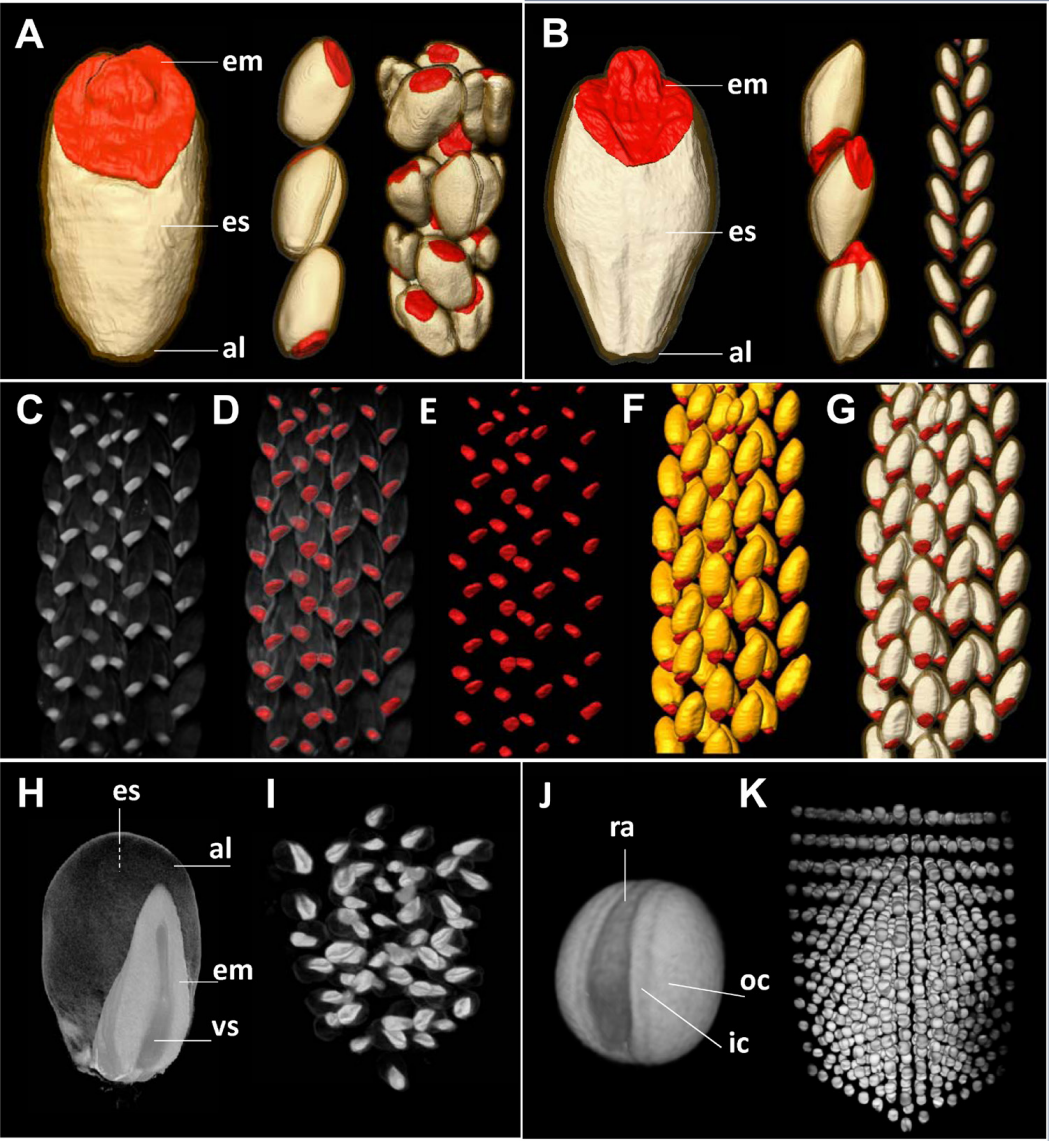
Multi-seed magnetic resonance imaging (MRI) of grains. (A) MRI-Seed-Wizard analysis of wheat grains using single, triplet, and multi-seed MRI (from left to right). (B) MRI-Seed-Wizard analysis of barley grains using single, triplet, and grains attached to the intact ear. (C–G) Survey of multiple intact ears of barley using MRI-Seed-Wizard demonstrates 3D MRI-based visualization of multiple grains attached to the multiple ears imaged simultaneously (C), the same image with embryo highlighted in red color (D), embryos only (E), embryos and aleurone (F), and embryos and endosperms (G). (H) High-resolution MRI of mature individual maize grain. (I) Multi-seed imaging of maize grains. (J) High-resolution MRI of mature individual rapeseed seeds. (K) Multi-seed imaging of rapeseed seeds. Abbreviations: al, aleurone; em, embryo; es, endosperm; ic, inner seed coat; oc, outer seed coat; ra, radicle; vs, vascular tissue.

Seed screening usually requires the analysis of a large number of seeds (as bulk) or seeds attached to the cereal ear. Hence, we next explored effective approaches to increase the number of exposed seeds at the same time. Assuming that convolutional operations were translation-invariant (i.e. the operations did not depend on the positioning and number of samples in space), we performed MRI scans of one, three, and 16 tightly packed wheat grain samples (9.4 T instrument), followed by automated segmentation using MRI-Seed-Wizard. As demonstrated in Fig. 6A, nnUnet worked with similar efficiency and reliable segmentation performance as for images with lower number of grains.

We used a similar approach for barley, scanning the entire intact ears with multiple grains attached (11.7 T instrument; Fig. 6B). Again, the nnUnet-based segmentation framework worked efficiently, producing segmentation results of nearly the same quality as the conventional test set with single- or triple-seed images. Hence, multiple intact ears with numerous grains attached could be reliably and quantitatively analysed simultaneously (Fig. 6C–G). Moreover, the images with barley ears had lower resolution than images from the training set of the grain segmentation model, but still resulted in acceptable quality, which indicates the robustness of the segmentation framework. No technical limitations exist, and only the quality of segmentation is a key point for the overall data quality in the Wizard workflow. We demonstrate here the application of the 11.7 T instrument for MRI multi-seed screening of maize (*Zea mays*) and rapeseed (*Brassica napus*). Simultaneous MRI screening of a number of seeds (~50 maize grains or ~750 rapeseed grains) provided suitable 3D images for segmentation (Fig. 6H–K).

## Discussion

Several studies have demonstrated the successful application of MRI in seed phenotyping (Fuchs *et al*., 2013; Rolletschek *et al*., 2015). While highly informative, MRI seed analysis still does not have a high-throughput and the complete potential of this technique is still not widely explored due to a lack of automated analysis of MRI images. In the current study, we developed a novel tool named MRI-Seed-Wizard that enabled automated semantic segmentation of volumetric MRI images and substantially facilitated grain analysis. The robustness of the segmentation algorithm, coupled with automated post-processing analysis, made it easy to implement, fast-to-run, and easily scalable. In combination with appropriate MRI protocols and hardware, the MRI-Seed-Wizard tool represents a significant advance in seed phenotyping technology.

### Advantages and relevance of MRI-Seed-Wizard in seed phenotyping

MRI-Seed-Wizard is based on machine learning and possesses unique properties that make it stand out from other seed screening methods. Unlike traditional machine learning applications, which require a huge amount of initial image data, our approach helped when starting with minimal datasets. The model began with a few initial 3D MRI images virtually dissected into planar slices. Increasing the number of initial training 2D slices using different rotational variants of the 3D objects helped improve the performance of the early model. Even in the early stages of model development, this tool significantly reduced the amount of manual segmentation work while constantly increasing the quality of the results. Next, we progressed from the early model that worked only with planar images to a model that worked with image stacks and finally to a model that processed 3D data under controllable conditions favorable for debugging and maintaining software and data. While manual segmentation of a single cereal grain takes around 2 h, MRI-Seed-Wizard processes the same grain in a few minutes, producing the segmented data with a comparable quality. MRI-Seed-Wizard can be used for analysis in 3D, providing a great advantage over methods based on visual observation, such as SmartGrain (Tanabata *et al*., 2012) or the MARVIN analyser (Gegas *et al*., 2010). MRI-Seed-Wizard is also advantageous over 3D structured light imaging screening (Huang *et al*., 2022) because MRI technology reveals the internal structures of the grains (Liu *et al*., 2024). In particular, MRI-Seed-Wizard is able to analyse a wide range of morphometric features, including the previously inaccessible characteristics, such as living embryo and endosperm, which give rise to a future plant and provide essential nourishment to sprouting seeds, respectively. MRI-Seed-Wizard can be used to automatically analyse the essential features of both of these seed structures, such as the shape, volume, and surface area. Another advantageous feature of MRI is the successful identification and visualization of lipids (Munz *et al.*, 2016), which is not attainable by X-ray CT or other 3D techniques (Schmidt *et al*., 2020; Liu *et al*., 2024). This feature enables the automated segmentation of lipid-enriched internal structures, such as the aleurone layer and the embryo (Figs 1, 2). Both these organs are most relevant for germination, and MRI-Seed-Wizard allows easy access to their structure, shape, and size. Our tool can thus enable targeted studies on these structures in the context of germination-related features. In contrast, X-ray CT often fails to identify tissues with similar electron density (Le *et al*., 2019). Distinguishing tissue types using X-ray might require additional seed treatment, such as the application of exogenous contrast agents. However, 3D data generation for MRI-Seed-Wizard does not involve any seed treatment or ionizing radiation, thereby eliminating potential risks to the seed’s meristem.

The output of MRI-Seed-Wizard can be linked with the data collected by other screening tools, providing data on biochemical composition. The present study demonstrates this aspect by screening and statistically analysing 30 grain traits determined for the NDM wheat population (Scott *et al*., 2021). The outcome revealed different levels of positive and negative correlations among the traits, most of which were not previously accessible (Table 1; Figs 3, 4). Upon demand or specific interest by breeders/seed scientists, the list of traits can be extended further by incorporating new algorithms. Overall, our novel approach paves the way for a comprehensive survey of seed traits related to the internal grain structure and the accumulation of specific storage compounds (such as lipids in the aleurone and the embryo or starch in the starchy endosperm). In addition, MRI-Seed-Wizard can be useful for dissecting the genetic background for the quantitative traits by analysing large bulks of genetic resources, such as bi-parental and MAGIC populations and panels of accessions for genome-wide association studies. The fast, accurate, and quantitative analysis of the internal seed structure opens the way for targeted improvement of seeds via breeding.

The versatility of MRI-Seed-Wizard extends its application to other cereal crops with minimal adjustments. This can be particularly important for assessing the phenotypes of mutant or transgenic grains, where the number of seeds, time of analysis, or rate of propagation is limited. We demonstrated how MRI-Seed-Wizard can define aberrations in the structure and/or filling of specific tissues of wild type and genetically manipulated grains of barley. The use of optical tools, like the MARVIN analyser, can severely compromise the precise survey of barley grains because these grains are covered with a husk (Radchuk *et al*., 2023). MRI-Seed-Wizard overcomes this problem, allowing a comprehensive comparative analysis of the grains (Fig. 6). Automatic segmentation data acquired here were in good agreement with manually performed measurements (Radchuk *et al.*, 2021), corroborating the lower endosperm and embryo volumes detected in the transgenic grains and additionally discovering lower aleurone volumes in these grains. Taken together, MRI-Seed-Wizard can be efficiently used for the analysis of other cereals where optical/NIRS-based analyses of grains are challenged by a husk (Keil *et al*., 2023). The portability of the wheat model to other cereals (such as oat and rye) might require some modifications, as demonstrated for barley in the current study, where adding a few samples of the new data to the training dataset was sufficient to overcome limitations.

### Limitations and prospects of the MRI-Seed-Wizard tool

MRI-Seed-Wizard can be adapted to different scenarios of segmentation and analysis, focusing on the seed domain or generalized for similar domains providing sufficient training data are available. The segmentation and post-processing aspects were independent, tractable, and adjustable. Every post-processing step could potentially be enhanced and customized. For example, the alignment of the objects relying on elongated seed shapes can be redesigned for round seeds using classical and DL algorithms.

Improving the throughput and quality of MRI image acquisition can significantly expand the application of MRI in seed screening. Some experimental strategies to increase sample throughput have proven effective in both experimental medicine and plant MRI. One of these strategies comprised elevating the number of simultaneously measured samples (Kotyk *et al*., 2005; Bamforth *et al*., 2012; Fuchs *et al*., 2013). The current study showed that the segmentation module of MRI-Seed-Wizard performed well for multi-seed screens with different resolution (Fig. 6) generated by different magnetic field strengths (9.4 T and 11.7 T). Previously, 1.5 T human MRI scanners have also been used for the simultaneous imaging of seeds (Kotyk *et al*., 2005). It provides scope for extending the application of MRI-Seed-Wizard. Appropriate MRI resolution, considering the seed size and available instrumentation, is primarily important in the operation of this tool. For example, moderate resolution was suitable for MRI of wheat, barley, maize, and rapeseed seeds (Supplementary Table S1). A much higher spatial resolution of 25 µm was required for the screening of submillimeter seeds of tobacco using a high field 17.6 T MRI instrument (Fuchs *et al*., 2013). Current progress in MRI technology allows for attaining comparable quality/resolution using 9.4 T instruments in combination with cryo-coils to improve sensitivity of signal detection. The MRI settings parameters can be further optimized to achieve higher resolution for a reasonable number of seeds, or alternatively, a more time-saving approach and efficient use of space inside the RF coil. It should be admitted that there are several ways to optimize the MRI procedure for the desired accuracy of measurements (McRobbie *et al*., 2006). MRI-Seed-Wizard can be adjusted to accommodate models other than wheat and barley seeds through the optimization of its components. It is important to note that the combined use of multi-seed MRI and MRI-Seed-Wizard has the potential to significantly enhance sample throughput, as evidenced in the cases of wheat and barley. However, it is imperative to account for the distinct characteristics of seeds across different species and to appropriately address these variations in their detectability via tailoring MRI-Seed-Wizard components. To this end, an important factor in facilitating seed screening is the use of modern instrumentation that allows a high homogeneity of the magnetic field for seed imaging (Borisjuk *et al*., 2023). Another approach to elevate sample throughput is the application of artificial intelligence in MRI to optimize image sequences and reduce computational time for reconstruction and post-processing (e.g. MR-fingerprinting) (Velasco *et al*., 2022), to enable faster scan acquisition and reconstruction of under-sampled data (for example, compressed sensing) (Chen *et al*., 2022). Recent advances in plant MRI and its computing power have helped achieve previously unattainable optimizations (Van As and Van Duynhoven, 2013; Borisjuk *et al*., 2023). MRI Seed Wizard provides reliable, automated segmentation for seed analysis, further advancing the field of MRI-based seed phenotyping.

## Supplementary data

The following supplementary data are available at *JXB* online.

Fig. S1. 3D MRI and lipid distribution of selected wheat cultivars.

Fig. S2. Consistency of dimensions with correlation analysis of length, width, depth (scatter plots on the left side), and respective difference plots according to the Bland–Altman method (Bland and Altman, 1986) on the right side.

Fig. S3. Aggregated difference plot of all measurements in relation to the mechanical measurements.

Fig. S4. Visual comparison of grain trait distribution to normal distribution as analysed by normality test of grain traits using quantile–quantile (QQ) plots (Marden, 2004).

Fig. S5. Magnetic resonance imaging (MRI) of mature barley grains and virtual dissections of main organs by manual segmentation.

Table S1. Setting MRI parameters for seed screening.

Table S2. Mean volumes of different organs in the grains of six wheat accessions after manual assignment and quantification.

Table S3. Mechanical measurement of grain dimensions and comparison with MRI Wizard output.

Table S4. Raw data for 30 phenotypic grain traits from 16 NDM founder lines.

Table S5. Statistical evaluation of wheat accessions based on MRI-Seed-Wizard.

Table S6. Raw data for the phenotypic traits of barley grains.

Video S1. Visualization of wheat grain segmentation results from models processed in 2D, 2.5D, and 3D formats.

erae408_suppl_Supplementary_Tables_S1-S2_Figures_S1-S5

erae408_suppl_Supplementary_Video_S1

erae408_suppl_Supplementary_Table_S3

erae408_suppl_Supplementary_Table_S4

erae408_suppl_Supplementary_Table_S5

erae408_suppl_Supplementary_Table_S6

## Data Availability

All data supporting the findings of this study are available within the paper and its supplementary data with codebase published online. Code and demonstration data are available at: https://github.com/akvilonBrown/mri-wizard.

## Abbreviations:

2D: two-dimensional;
3D: three-dimensional
CT: computed tomography
cv.: cultivar
DL: deep learning
MRI: magnetic resonance imaging
NMR: nuclear magnetic resonance
PCA: principal component analysis
TD-NMR: time domain NMR
