# Supplementary material for: MRI-Seed-Wizard: combining deep learning algorithms with magnetic resonance imaging enables advanced seed phenotyping
Source: J Exp Bot. 2024 Oct 9;76(2):393–410. doi: 10.1093/jxb/erae408 (PMC11714760; doi:10.1093/jxb/erae408)
Supplement: erae408_suppl_Supplementary_Tables_S1-S2_Figures_S1-S5 [file erae408_suppl_supplementary_tables_s1-s2_figures_s1-s5.pdf]

Table S1. Setting MRI parameters for seed screening

Sample	Device	Probe head	FOV [mm]	Resolution [µm]	Repetition Time TR [ms]	Echo Time TE [ms]	Average Time [min]
**Wheat (single grain)**	400 MHz	1H cryocoil 5mm	9.2x4.2x4.2	50 isotropic	750	7.6	88
**Wheat (triplet)**	400 MHz	1H cryocoil 10mm	20x4.5x4.5	80 isotropic	900	5.8	47
**Wheat (multi-seed)**	400 MHz	1H cryocoil 10mm	25x9x9	90 isotropic	1000	6.4	166
**Barley (single grain)**	400 MHz	1H cryocoil 5mm	9.2x4.4x4.4	40 isotropic	750	7.7	151
**Barley (triplet)**	400 MHz	1H cryocoil 10mm	20x4.5x4.5	80 isotropic	1000	7.2	52
**Barley (multi-seed)**	500 MHz	1H 66mm	23x29x60	300 isotropic	700	2.6	224
**Maize (multi-seed)**	500 MHz	1H 66mm	40x40x60	400 isotropic	750	3.0	187
**Rapeseed (multi-seed)**	500 MHz	1H 66mm	52x24 x 24	100 isotropic	700	7.0	672

When working with similar samples, measurement time can be significantly reduced without changing resolution by simply using a smaller field of view. For example, the average measurement time for several canola seeds with a large field of view of 51 x 32 x 32 (and TR = 800 ms) is 1365 min, while with a field of view of 52 x 24 x 24 (and TR = 700 ms) it is only 672 min for the same isotopic resolution of 100 microns. The shape/size of the seed should be considered when providing efficient use of space within the RF coil. Alternatively, the very high resolution is not always necessary. In such a case, time savings can be achieved by a reasonable resolution reduction. For example, setting the resolution to 120 microns instead of 100 microns (at the same FOV 52 x 24 x 24) will reduce the time to measure the same set of seeds by 206 min.

Table S2. Mean volumes of different organs in the grains of six wheat accessions after manual assignment and quantification. Data are given as means +/- standard deviation (n = 3).

Accession	Monolith, mm^3	Embryo volume, mm^3	Aleurone volume, mm^3	Endosperm volume, mm^3
Flair	47.47±1.33	3.87±1.01	12.59±0.55	31.01±1.42
Nirvana	45.49±2.07	2.88±0.12	11.60±1.12	31.00±1.04
Nadobna	29.38±3.68	2.48±0.67	8.87±0.94	18.04±2.96
Prinz	45.07±0.57	4.28±0.71	11.68±0.41	29.11±0.43
Toras	42.22±3.29	3.74±0.21	11.28±0.83	27.21±2.49
Welford	38.22±4.97	3.31±0.08	10.68±0.77	24.24±4.20

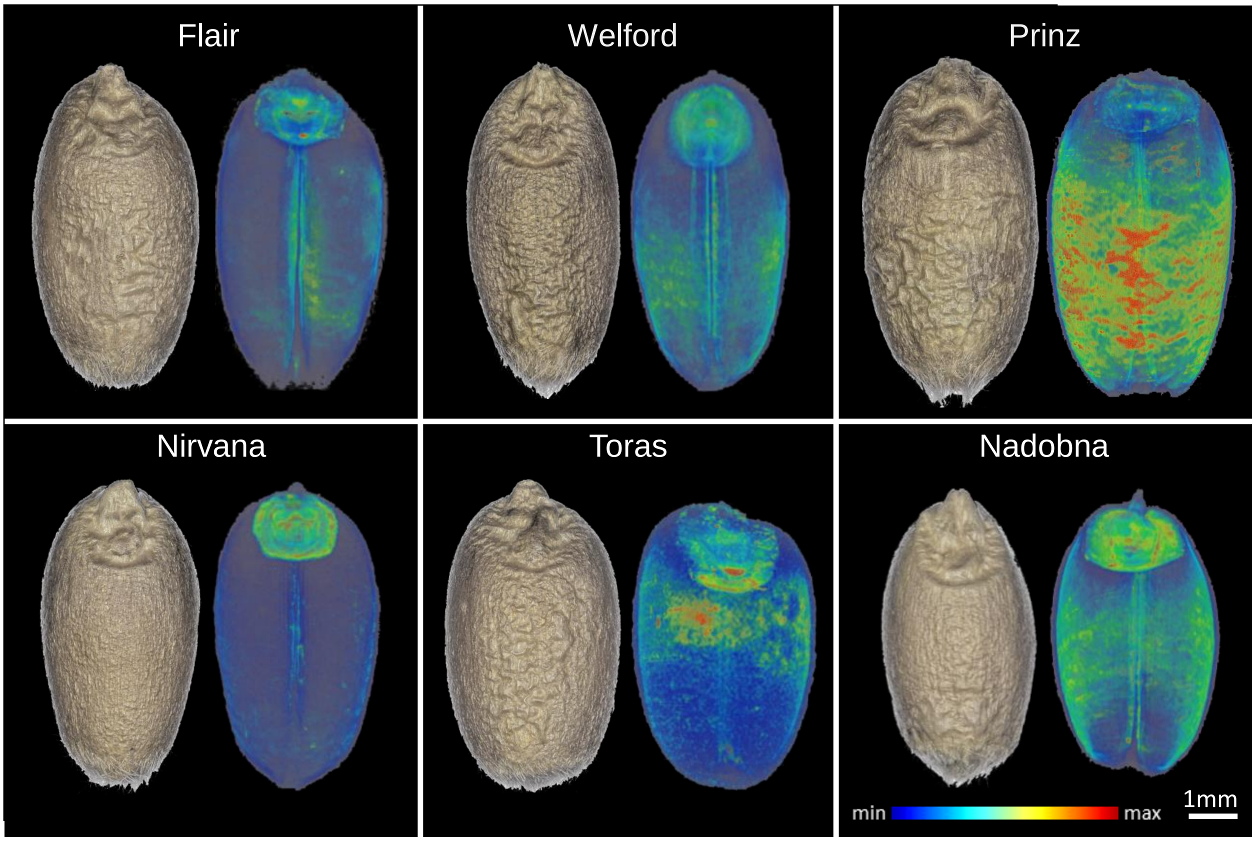

Figure S1. 3D MRI (left) and lipid distribution (right) of selected wheat cultivars. The lipid levels are color-coded. Bar = 1 mm.

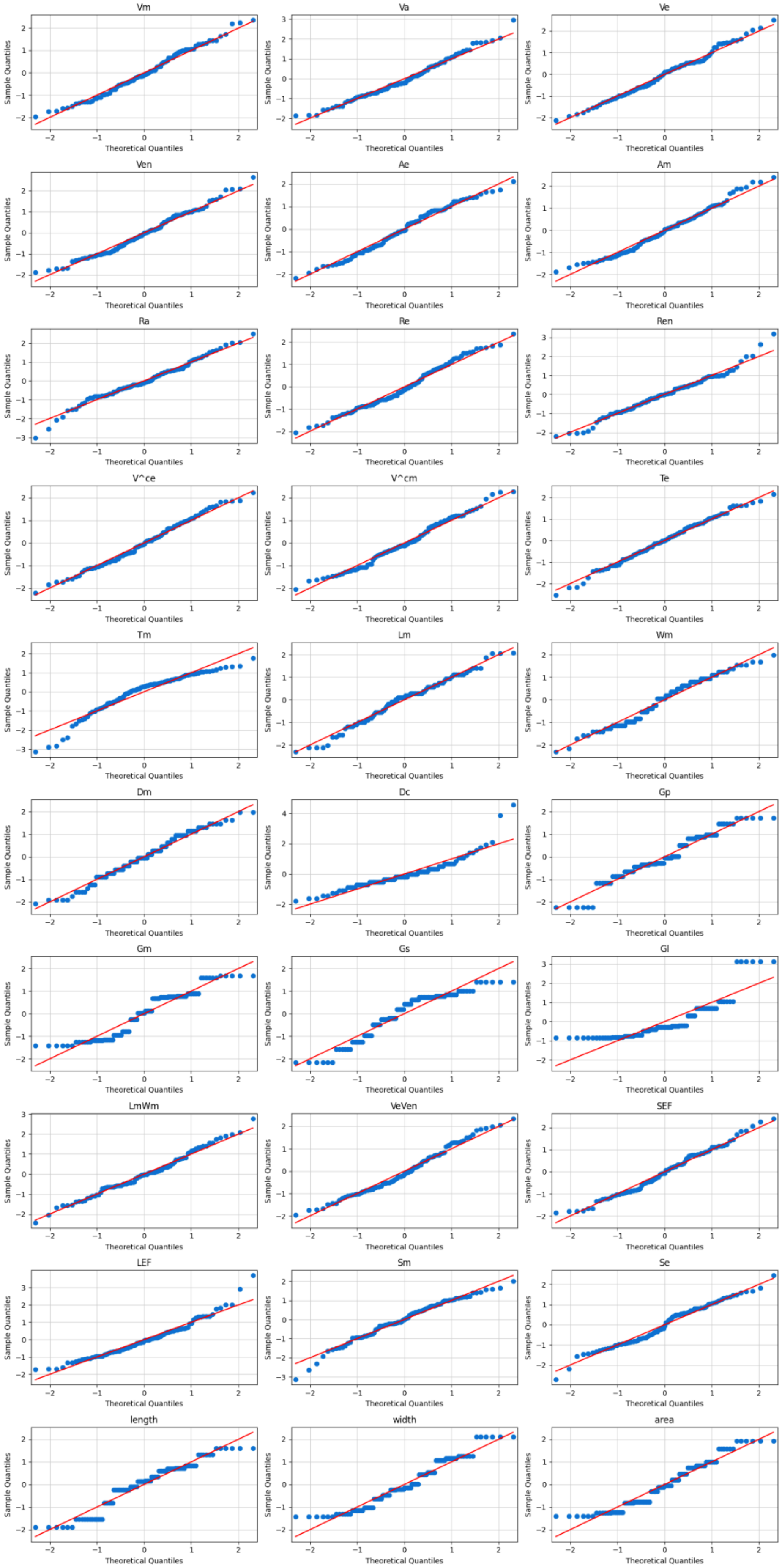

Figure S2. Visual comparison of grain trait distribution to normal distribution as analyzed by normality test of grain traits using Quantile Quantile (QQ) plots (Marden, 2004). Normal distribution should approximate the identity line y = x, shown in red. Glossary of abbreviations and explanations of grain traits are provided in Table 1.

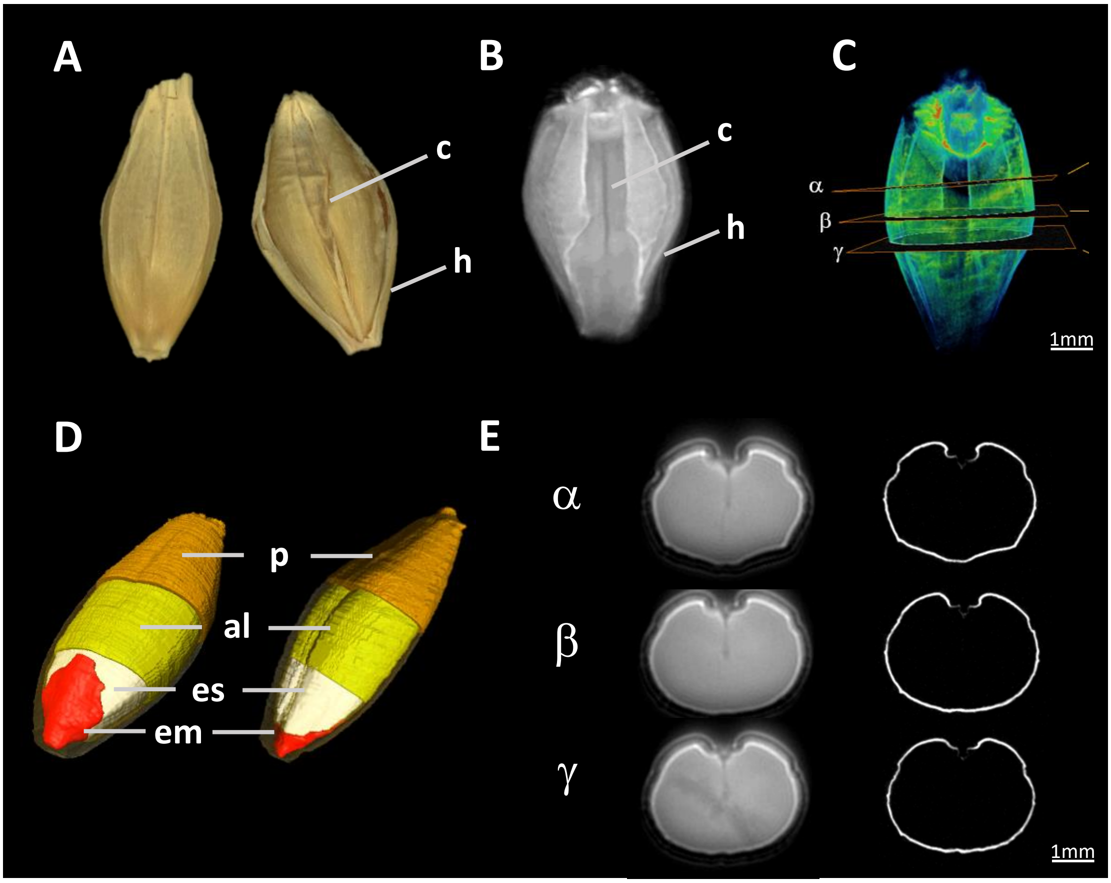

Figure S3. Magnetic Resonance Imaging (MRI) of mature barley grains and virtual dissections of main organs by manual segmentation.

(A) Optical image of the mature barley grain. (B) 3D visualization of individual grain by ^{1}H-MRI. (C) Localization of lipid in the same grain by MRI, lipid levels are color-coded. Positions for the virtual cross sections shown in (E) are labeled by α, β and γ. (D) MRI-based manual segmentation of the main organs of barley showing grain's internal structures. (E) Virtual cross sections of the grain showing the endosperm (bright signal, left panel) and aleurone layer (right panel). Bars = 1 mm. Abbreviations: al, aleurone; c, crease; em, embryo; es, endosperm; h, husk; p, pericarp.

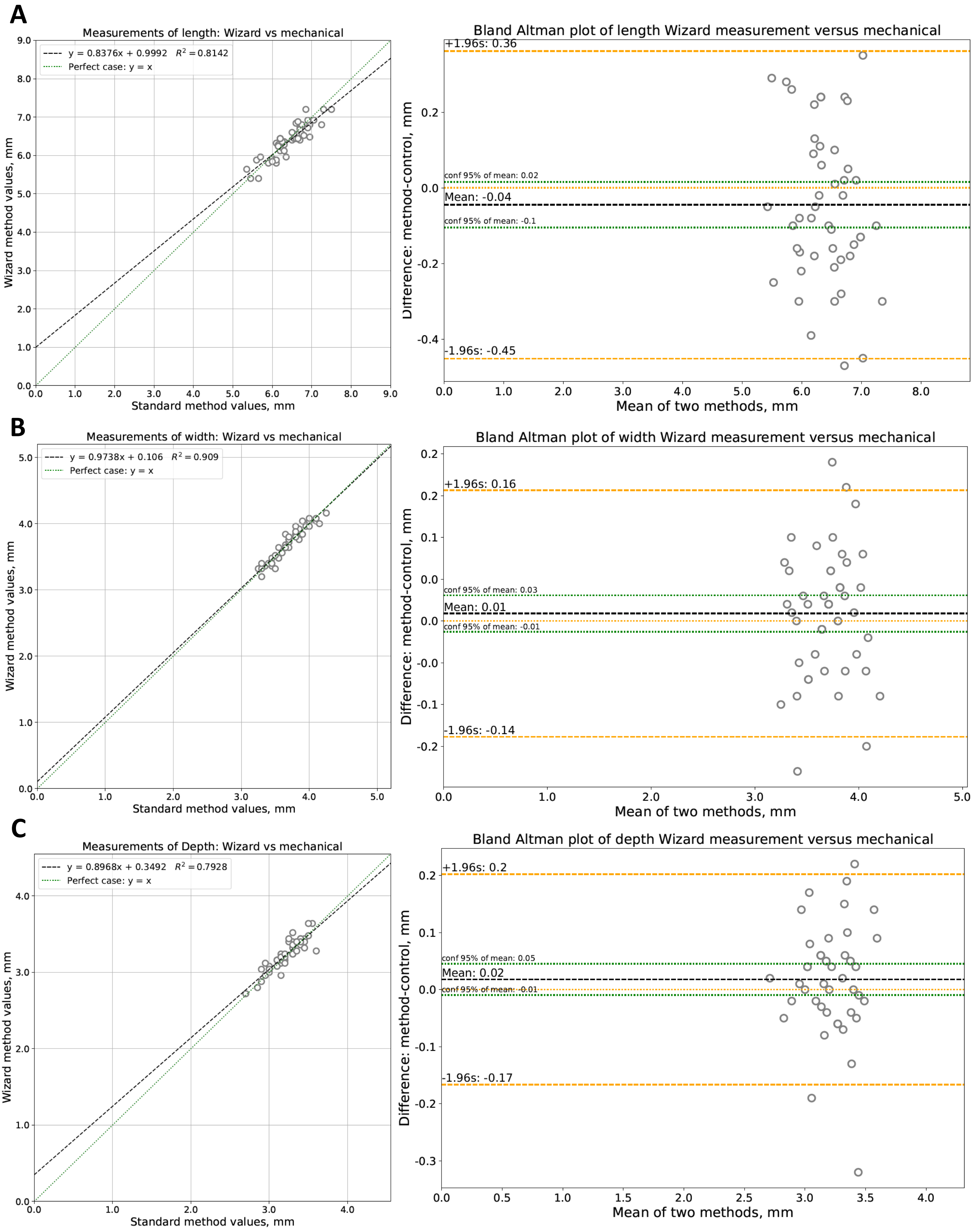

Figure S4. Consistency of dimensions with correlation analysis of length (A), width (B) and depth (C). The scatter plots shown on the left side and respective difference plots according to the Bland-Altman method (Bland and Altman, 1986) on the right side.

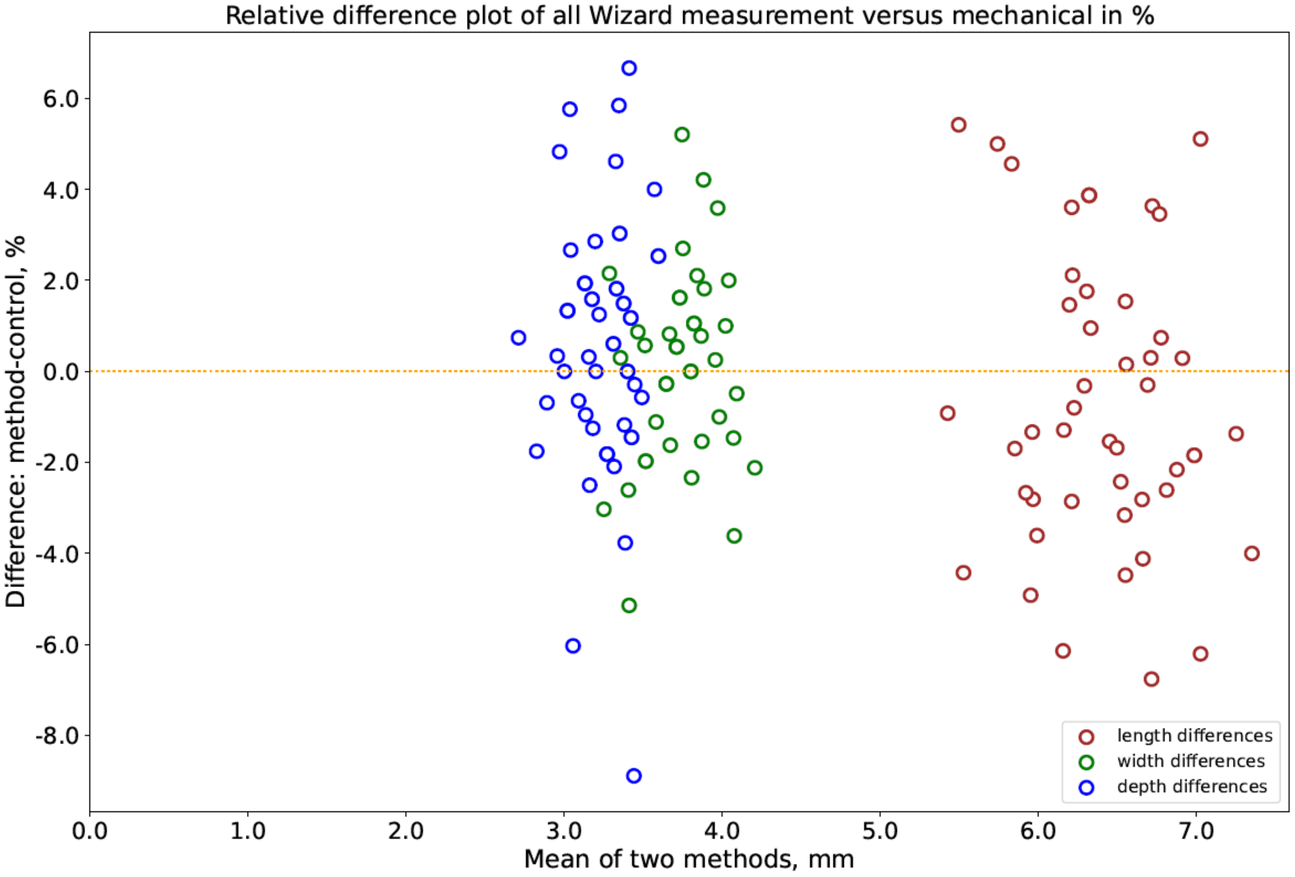

Figure S5. Relative differences of all dimension measurements in relation to the mechanical measurements. The difference is expressed as a percentage of mechanical measurements.
